# CT Detection of an Anomalous Left Circumflex Coronary Artery from Pulmonary Artery (ALXCAPA) in 81-Year-Old Female Patient

**DOI:** 10.3390/jcm12010226

**Published:** 2022-12-28

**Authors:** Marian Pop, Zsófia Kakucs, Simona Coman

**Affiliations:** 1 ME1 Department, George Emil Palade University of Medicine, Pharmacy, Science and Technology of Targu Mures, 540142 Târgu Mureș, Romania; 2 Emergency Institute for Cardiovascular Disease and Transplant of Targu Mures, 540136 Târgu Mureș, Romania; 3 Mures County Clinical Emergency Hospital, 540136 Târgu Mureș, Romania

**Keywords:** coronary vessels anomalies, anomalous origin of the left circumflex coronary artery from the pulmonary artery, ALXCAPA, coronary CT angiography

## Abstract

Background: The left circumflex coronary artery from the pulmonary artery is a very rare congenital anomaly with few cases described, so far, worldwide. Case report: An 81-year-old female presented complaining of dyspnea. The transthoracic echocardiogram revealed severe degenerative aortic stenosis in addition to a hypertrophied left ventricle with normal function and no wall motion abnormalities. As part of the pre-TAVI planning, she underwent a CT examination, which revealed an anomalous left circumflex artery originating from the right pulmonary artery. The case is currently being managed conservatively. Conclusion: The presented congenital coronary anomaly is, to our knowledge, the first to be described in the literature in this age group (80+).

## 1. Introduction

The classic configuration of coronary tree anatomy consists of the left main coronary artery (LMCA) and the right coronary artery (RCA), arising from the left and right coronary sinuses, respectively, in the aortic root. Typically, the LMCA is a short common stem, which bifurcates into the left anterior descending (LAD) artery and the left circumflex (LCX) artery [1].

The prevalence of coronary artery anomalies (CAAs) shows a wide variation between 0.3% and 2% in the general population [2]. CAAs are typically found incidentally during coronary angiography or autopsy studies. Considering that many hemodynamically insignificant cases remain undiagnosed, the actual prevalence rate is probably higher. Anomalous origins of the coronary arteries from the pulmonary trunk are relatively rare [3]. The left coronary artery from the pulmonary artery (ALCAPA), also known as Bland-White-Garland syndrome, is a well-recognized cardiovascular malformation affecting 1 in 300,000 births and constitutes 0.25–0.5% of total congenital heart defects [4,5,6,7].

The anomalous LCX coronary artery from the pulmonary artery (ALXCAPA) can be considered an extremely rare variant of the ALCAPA, with the first adult case documented in 1992 [8] and only 47 cases reported worldwide by 2021 [9]. The presence of an ALXCAPA in adult patients without congenital heart disease is extremely uncommon. Since 1992, only few cases have been described, with stable angina being the most frequent presenting symptom. While the ALCAPA is frequently identified in infancy due to ischemic symptoms related to flow changes consecutive to pulmonary artery pressure decrease [10,11], the ALXCAPA is usually discovered in adults [9,12], appearing concomitantly with other congenital symptomatic or life-threatening cardiac defects. It is often accompanied by aortic coarctation, patent ductus arteriosus, and subaortic or pulmonary valve stenosis [13].

The ALXCAPA identification in the more senior population has asked [14,15] for a reconsideration of its impact on the patient’s prognosis.

## 2. Case Description

We reported a case of an 81-year-old female patient, diagnosed five years before with aortic stenosis and arterial hypertension. She was in good health, with two adult children and minor non-cardiovascular complaints (lumbar disc herniation) at the time.

Three months before a CT examination, she was referred again to the cardiology outpatient clinic due to a worsening of the clinical status with palpitations and fatigability.

At the time of the examination, the patient’s clinical condition was stable, with a heart rate of 52 beats per minute and a blood pressure of 180/100 mmHg. A systolic murmur of grade 3/6 was found during the clinical examination, but there was no pulmonary or systemic congestion.

Her ECG showed a sinus rhythm with pressure overload changes, large R-waves, and end-phase diffuse ischemic changes.

The echocardiography showed concentric left ventricular hypertrophy with no cardiac wall motion abnormalities and an ejection fraction of 58%. The right ventricle function was normal, and there were no signs of pulmonary arterial hypertension. The aortic valve was intensely hyperechoic, with a maximum gradient of 100 mmHg and an average of 23 mmHg, and the diagnosis of severe degenerative aortic stenosis was set. The patient was referred to the cardiovascular radiology department for a CT evaluation prior to the trans-catheter aortic valve implantation (TAVI).

In accordance with the local pre-TAVI protocol, the examination included a native coronary calcium score assessment, an ECG-gated thoracic Angio CT examination with multiphasic contrast agent administration, and a non-ECG-gated Angio CT examination of the abdomen and pelvis. Minor breathing artifacts were noted.

Extensive coronary artery calcifications were discovered in systemically supplied coronary arteries, but they were very mild in the circumflex coronary artery, which originated from the pulmonary artery (Supplementary Video S1). The total Agatston score was 4322.72, placing her in the 99th percentile according to age, gender, and ethnicity; however, the LCX calcification represented only 6.56 of those.

A normal left main coronary artery was found to arise from the left coronary sinus, giving rise only to a dilated left anterior descending (LAD) artery (5.9 mm). The dilation of the LAD was evident at the level of the diagonals (4 mm) and septal perforator branches (2 mm) as well.

**Figure 1:**
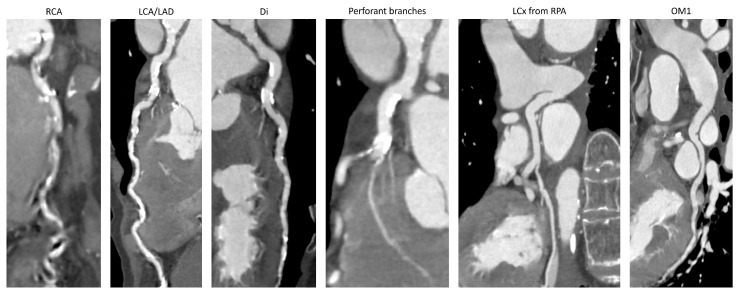
Curved reformat of the coronary arteries from their ostia. There were extensive calcification on the branches, starting from the systemic circulation (RCA, LCA/LAD, and diagonals), and only trivial calcification spots in the left circumflex coronary artery and in the obtuse marginal branch. The coronary arteries are enlarged, with perforant branches from the LAD reaching 2 mm in diameter. RCA—right coronary artery; LCA—left coronary artery; LAD—left anterior descending coronary artery; Di—diagonal branch; LCx—left circumflex coronary artery; OM1—first obtuse marginal.

**Figure 2:**
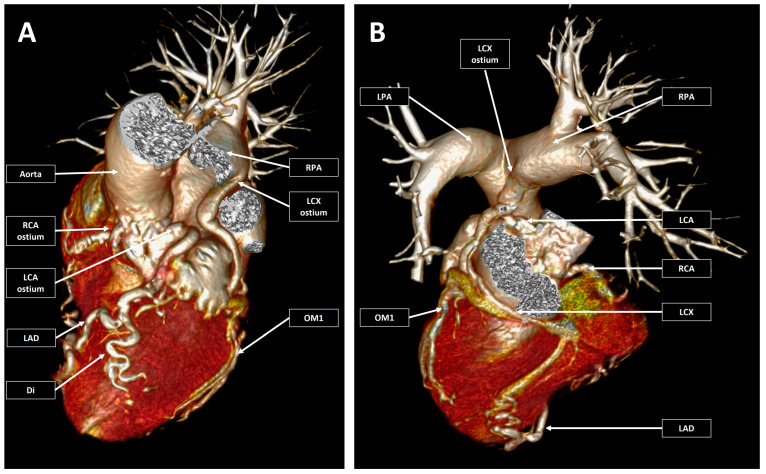
3D virtual rendering technique reconstruction of the heart, main vessels, and coronary arteries. Pannel (**A**) (left) Left/cranial double oblique anterior reconstruction with the main pulmonary artery and the left pulmonary artery removed from the image. Pannel (**B**) (right) Right/cranial double oblique posterior reconstruction with the removal of the atria, ascending aorta, aortic arch, and descending aorta. The coronary arteries are enlarged and had a tortuous course. There were both left and right coronary arteries emerging from the corresponding coronary sinuses, and a circumflex coronary artery emerged from the right pulmonary artery. RCA—right coronary artery; LCA—left coronary artery; LAD—left anterior descending coronary artery; Di—diagonal branch; LCx—left circumflex coronary artery; OM1—first obtuse marginal; LPA—left pulmonary artery; RPA—right pulmonary artery.

The enlarged left circumflex coronary artery (LCX; 3.5 mm) arose from the inferior aspect of the right pulmonary artery (Figure 1 and Figure 2 and Supplementary Video S2) and gave two thin branches (retroatrial and lateroatrial). It was enlarged up to 8 mm, with a large obtuse marginal, and ended as a posterolateral branch.

The right coronary artery (RCA) was codominant, originated in the typical location, and ended as the posterior descending artery with a serpiginous course.

While the overall coronary circulation was dilated, no definite RCA−LCA collaterals were observed; however, the presence of atypical LCX branches and the enlarged intramyocardial branches was indicative of an established network of collaterals draining eventually into the RPA.

The left ventricle appeared with a normal volume (75.6 mL, 508.5 mL/m^2^), with thickened walls up to 17.8 mL in the septum and 14.6 mm in the lateral wall.

No congenital malformations have been identified. 

The main pulmonary artery was in normal size (26 mm), but both branches were enlarged, with the right pulmonary artery displaying a fusiform aneurysmal aspect reaching 29 mm in the region of the LCX ostium. The normal right-to-left ventricle diameter ratio and the normal arterio-bronchial ratio also implied the lack of pulmonary hypertension.

A general thoraco-abdominal assessment showed minor TB sequelae, diffuse atheromatous plaques, and diffuse bone demineralization, but no further lesions.

The TAVI procedure has been reconsidered, with the patient currently undergoing medical treatment, with mild improvement of the symptoms at the last clinical re-evaluation.

## 3. Discussion

We reported the case of an 81-year-old female patient who was diagnosed with ALXCAPA following an Angio CT examination.

This type of anomaly may cause hemodynamic impairment. In case of the ALXCAPA, the posterolateral wall of the left ventricle (LV) is perfused by the abnormally arising coronary artery providing relatively low perfusion pressure and deoxygenated blood. In case of well-developed collateral circulation and decreased pulmonary vascular resistance, steal of blood flow will occur, a retrograde filling of the LCX from LAD artery and the RCA circulation (left-to-right shunt). Additionally, due to volume overload via left-to-right shunt, pulmonary hypertension may occur. Pulmonary hypertension can minimize the shunt from the LCX to pulmonary arteries and maintain the myocardial perfusion at a sufficient level. The hypoperfusion of myocardium will be prevented by a well-collateralized and pressured system, enabling survival to adulthood. However, the left-to-right shunt will induce a chronic increase in the LV preload, which will cause progressive dilatation of the LV and deterioration of the LV function [16].

Clinical presentation and survival depend upon the degree of collateralization from the two other coronary arteries. Adult patients may initially present with new-onset stable angina, palpitation, dyspnea, abnormal ischemic changes on electrocardiogram, or wall motion abnormalities observed on echocardiography [13]. Sudden cardiac death can occur after myocardial ischemia during physical activity, stress, or ventricular arrhythmias triggered by a previously developed scar tissue [17]. The symptoms of the ALXCAPA may present in different periods of life; however, some patients remain asymptomatic with the anomaly being identified incidentally during diagnostic procedures [18]. We hypothesize that our patient remained asymptomatic throughout her life and reached the age of 81 due to the development of adequate coronary collateralization and the relatively small area of myocardium supplied by the LCX artery.

Invasive coronary angiography (ICA), which is widely available and considered the gold standard diagnostic method, provides visualization of collateral vessels and enables assessing the amount of retrograde flow from collateral vessels [14]. Moreover, in case of CAAs, ICA should be performed to exclude additional stenosis. The coronary computed tomography angiography (CCTA) has been shown to be a promising substitute for cardiac catheterization as a non-invasive imaging method of identifying abnormal coronary origin and course [19]. In some cases, the precise origin of aberrant coronary vessels could not be identified by ICA. In such cases, CCTA and cardiac magnetic resonance imaging are recommended, which allow unambiguous identification of coronary origins [13].

CT allows for the quantification of calcified plaques, which we found to be extensive for systemic-supplied coronaries and only trivial in the branch connected to pulmonary circulation. In previously reported ALXCAPA cases [12], we found no mention of such extensive calcification and we believe that their asymmetrical distribution may be attributable to the variations in the local pulse pressure; however, since survival in untreated ALCAPA patients is uncommon beyond the age of 50, there are little data on the amount of calcified plaques in very elderly patients with coronary anomalies.

In diagnosing the ischemic effects of anomalous coronary arteries, stress testing (echocardiogram, magnetic resonance imaging, or CT stress perfusion) has proven useful [20], demonstrating perfusion deficit in the territory affected. Our CT examination, conducted with the patient at rest, did not reveal any structural changes in the myocardium that might indicate perfusion deficit or long-term ischemic damage.

There are no consensus defining the standard management and operative techniques for patients with the ALXCAPA [15]. Although the latest guidelines recommend surgical treatment for asymptomatic ALCAPA, there is no specific guidance on variants such as the ALXCAPA. The clinical course of the ALXCAPA may not always be favorable, and some patients need surgical treatment in early infancy. In the ALCAPA, the surgical correction is mandatory, when symptoms are attributed to ischemia [14]. Surgical treatment options include reimplantation of the anomalous vessel into the aorta [17,21], ligation, or ligation with aorto-coronary bypass. Even if in many previous cases the surgical technique of choice was ligation followed by bypass [5], the current surgical techniques focus, whenever possible, on the anatomical reimplantation, regardless of the patient age [12], with overall good results [22]. Although cardiovascular surgery has been mostly used, the conservative approach may be an option in some circumstances. Although cardiovascular surgery has been mostly used, the conservative approach may be an option in some circumstances [15].

There is limited experience on the management of concurrent ALCAPA and aortic valve disfunction [23], with surgical correction in such an age group being uncertain to provide a survival benefit. Transcatheter replacement of the aortic valve might improve aortic stenosis symptoms; however, in a patient that has been balancing such conditions for a long time, a change in the hemodynamic status can provide unpredictable results.

## 4. Conclusions

We described a rare case of the isolated left circumflex coronary artery from the right pulmonary artery. This is the first case report of a patient with ALXCAPA in the age group of 80+, and it shows extensive calcification in systemic-supplied coronary arteries and trivial calcification spots in pulmonary-connected coronary arteries.

## Data Availability

Not applicable.

## Supplementary Materials

The following supporting information can be downloaded at: https://www.mdpi.com/article/10.3390/jcm12010226/s1, Video S1: Calcium score examination; Video S2: 3D virtual rendering technique reconstruction of the heart, main vessels, and coronary arteries.

## References

[1] Angelini P. Curicculum in Cardlology Normal and Anomalous Coronary Arterys: Definitions and Classification.

[2] Namgung J., Kim J.A. (2014). The Prevalence of Coronary Anomalies in a Single Center of Korea: Origination, Course, and Termination Anomalies of Aberrant Coronary Arteries Detected by ECG-Gated Cardiac MDCT. BMC Cardiovasc. Disord..

[3] Gentile F., Castiglione V., de Caterina R. (2021). Coronary Artery Anomalies. Circulation.

[4] Sadoma D., Valente C., Sigal A. (2019). Anomalous Left Coronary Artery From The Pulmonary Artery (ALCAPA) as a Cause of Heart Failure. Am. J. Case Rep..

[5] Liu B., Fursevich D., O’dell M.C., Flores M., Feranec N. (2016). Anomalous Left Circumflex Coronary Artery Arising from the Right Pulmonary Artery: A Rare Cause of Aborted Sudden Cardiac Death. Cureus.

[6] Hauser M. (2005). Congenital Anomalies of the Coronary Arteries. Heart.

[7] Angelini P. (2007). Coronary artery anomalies: An entity in search of an identity. Circulation.

[8] Garcia C.M., Chandler J., Russell R. (1992). Anomalous Left Circumflex Coronary Artery from the Right Pulmonary Artery: First Adult Case Report. Am. Heart J..

[9] Ziermann F.K. (2021). Fehlkonnektierte Koronararterien Zur Pulmonalarterie—Primäre Befunde Und Anatomische Besonderheiten. Ph.D. Thesis.

[10] Walsh M.A., Duff D., Oslizlok P., Redmond M., Walsh K.P., Wood A.E., Coleman D.M. (2008). A Review of 15-Year Experience with Anomalous Origin of the Left Coronary Artery. Ir. J. Med. Sci..

[11] Peña E., Nguyen E.T., Merchant N., Dennie C. (2009). ALCAPA Syndrome: Not Just a Pediatric Disease. Radiographics.

[12] Guenther T.M., Sherazee E.A., Gustafson J.D., Wozniak C.J., Brothers J., Raff G. (2020). Anomalous Origin of the Circumflex or Left Anterior Descending Artery From the Pulmonary Artery. World J. Pediatr. Congenit. Heart Surg..

[13] Korosoglou G., Ringwald G., Giannitsis E., Katus H.A. (2008). Anomalous Origin of the Left Circumflex Coronary Artery from the Pulmonary Artery. A Very Rare Congenital Anomaly in an Adult Patient Diagnosed by Cardiovascular Magnetic Resonance. J. Cardiovasc. Magn. Reson..

[14] Cabrera-Huerta S.P., Martín-Lores I., Cabeza B., Gómez de Diego J.J., Pérez de Isla L., Vilacosta I., Pozo-Osinalde E. (2020). Circumflex Artery Arising From the Pulmonary Artery: Always a Malignant Coronary Anomaly?. JACC Case Rep..

[15] Separham A., Aliakbarzadeh P. (2012). Anomalous Left Coronary Artery from the Pulmonary Artery Presenting with Aborted Sudden Death in an Octogenarian: A Case Report. J. Med. Case Rep..

[16] Cambronero-Cortinas E., Moratalla-Haro P., González-García A.E., Oliver-Ruiz J.M. (2020). Case Report of Asymptomatic Very Late Presentation of ALCAPA Syndrome: Review of the Literature since Pathophysiology until Treatment. Eur. Heart J. Case Rep..

[17] Vergara-Uzcategui C.E., Urquiza R.V., Salinas P., Nunez-Gil I.J. (2021). Anomalous Origin of Left Circumflex Artery from the Right Pulmonary Artery of an Adult. REC Interv. Cardiol..

[18] Al-Muhaya M.A., Syed A., Najjar A.H.A., Mofeed M., Al-Mutairi M. (2017). Anomalous Origin of Circumflex Coronary Artery from Right Pulmonary Artery Associated with Atrial Septal Defect. J. Saudi Heart Assoc..

[19] Kakucs Z., Heidenhoffer E., Pop M. (2022). Detection of Coronary Artery and Aortic Arch Anomalies in Patients with Tetralogy of Fallot Using CT Angiography. J. Clin. Med..

[20] Laflamme E., Alonso-Gonzalez R., Roche S.L., Wald R.M., Swan L., Silversides C.K., Thorne S.A., Horlick E.M., Benson L.N., Osten M. (2021). Anomalous Origin of a Coronary Artery from the Pulmonary Artery Presenting in Adulthood: Experience from a Tertiary Center. Int. J. Cardiol. Congenit. Heart Dis..

[21] Bolognesi R., Alfieri O., Tsialtas D., Manca C. (2003). Surgical Treatment of the Left Circumflex Coronary Artery from the Pulmonary Artery in an Adult Patient. Ann. Thorac. Surg..

[22] Fudulu D.P., Dorobantu D.M., Taghavi M., Sharabiani A., Angelini G.D., Caputo M., Parry A.J., Stoica S.C. (2015). Outcomes Following Repair of Anomalous Coronary Artery from the Pulmonary Artery in Infants: Results from a Procedure-Based National Database. Open Heart.

[23] Yong L. (2021). Asymptomatic Adult Type ALCAPA Syndrome Coexisting with Bicuspid Aortic Valve- A Case Report. J. Cardiol. Cardiovasc. Res..

